# Olfactory perception and wellbeing across hormonal contraceptive users and menstrual cycle phases

**DOI:** 10.3389/fnhum.2026.1646597

**Published:** 2026-02-03

**Authors:** Patrícia Maria Rodrigues Gonçalves, Bruna Cestari de Azevedo, Denilson Fomin, Raul Galliano Galeazzo, César Antônio Veiga, Natália Queiroz Rezende, Isadora Pozzetti Siba, Raphaela Goncalves Barros Pascarelli, Gustavo Dieamant, Sérgio Podgaec, Camila Hernandes Pinheiro

**Affiliations:** 1 Hospital Israelita Albert Einstein, São Paulo, Brazil; 2 Grupo Boticário, São José dos Pinhais, Brazil

**Keywords:** emotional wellbeing, menstrual cycle, olfactory performance, oral contraceptives, sex hormones

## Abstract

Hormonal fluctuations are known to influence olfactory performance, but the effects of oral contraceptive (OC) use remain unclear. This study examined 98 women aged 18–40, including OC users and naturally cycling women, using the Sniffin’ Sticks test—a standardized assessment of olfactory threshold, discrimination, and identification—and self-report measures of life satisfaction and subjective happiness to capture emotional factors linked to olfaction. All participants were evaluated at two standardized time points: for naturally cycling women, during the periovulatory and luteal phases; for OC users, during the corresponding active and inactive phases of the pill cycle; and for continuous users, at matched intervals. OC users were categorized by hormonal formulation (ethinylestradiol 0.02 mg, ethinylestradiol 0.03 mg, estradiol valerate, or progestogen-only pills) and regimen (cyclical or continuous). No overall differences in olfactory performance were found between OC users and non-users. Exploratory analyses indicated that women using estradiol valerate-based oral contraceptives tended to show higher threshold and TDI scores compared to other regimens, a pattern that warrants further investigation given the limited sample size (*n* = 4). Lower olfactory performance was also associated with progestogen-only formulations and continuous OC use, and longer OC use duration was negatively associated with threshold sensitivity. Across all groups, better olfactory performance was related to higher subjective wellbeing in both OC (*n* = 47) and NOC (*n* = 51) groups. By examining specific contraceptive types and incorporating emotional wellbeing as a variable, this study contributes new insights into the complex relationships between hormonal status and sensory-emotional functioning. Overall, these preliminary findings suggest that contraceptive formulation, regimen, and emotional state may be linked to variability in olfactory processing, highlighting underexplored associations that may inform future research on hormonal contraception and sensory-emotional functioning.

## 1 Introduction

The role of sex steroid hormones—particularly estradiol and progesterone—in modulating physiological and behavioral processes across the menstrual cycle has been extensively documented (Veldhuijzen et al., 2013; Raghunath et al., 2015; Catenaccio et al., 2016; Meeker et al., 2020). These hormonal oscillations, especially the periovulatory peak in estradiol, appear to be associated with variations in olfactory function in women (Farage et al., 2008; Kanageswaran et al., 2016; Kollndorfer et al., 2016). During ovulation—marked by the luteinizing hormone (LH) surge and estradiol zenith—numerous physiological and perceptual changes occur (Fehring, 2002; Parlee, 1983), many of which are attenuated in women using combined oral contraceptives (OCs) due to suppression of endogenous hormonal fluctuations (Elkind-Hirsch et al., 1992; Renfro and Hoffmann, 2013).

Diverse studies have examined the possible associations between hormonal fluctuations during the menstrual cycle and the use of OCs on olfactory perception. In general, findings suggest that olfactory performance may vary depending on the menstrual phase and contraceptive use, although results remain inconclusive. For instance, some studies found that women in the periovulatory phase exhibit greater sensitivity to social odors such as androstenone and musk compared to women using OCs, an effect associated with the estrogen peak at mid-cycle (Lundström et al., 2006; Nováková et al., 2014). In contrast, opposite results were observed when testing environmental odors, with higher identification scores during the luteal phase (Bogdan et al., 2021). Hormonal oscillations seem to affect not only sensitivity but also odor discrimination: women not using OCs showed reduced discrimination performance in the luteal phase, while users experienced this decrease during the hormone-free interval (Endevelt-Shapira et al., 2020). The use of OCs may attenuate natural fluctuations, as shown by Caruso et al. (2001), who reported that olfactory sensitivity during the cycle was reduced and stabilized after initiating OC use. Additionally, Derntl et al. (2013) found positive correlations between olfactory performance, menstrual cycle phase, and duration of contraceptive use, and Kollndorfer et al. (2016) observed better olfactory scores in women using low-dose ethinylestradiol (0.020 mg) compared to those using higher doses (0.030 mg). However, not all studies support these associations; Schaefer et al. (2021), for example, found no significant differences in threshold, discrimination, or identification scores between OC users and non-users.

Collectively, these findings reveal a complex and sometimes contradictory picture: olfactory performance is subject to modulation by hormonal status, but effects are influenced by factors such as odor type, hormonal dose, cycle phase definition, and methodological differences across studies. This variability suggests that the relationship between hormonal fluctuations and olfactory perception is not uniform, and further research is needed to clarify the conditions under which such associations may emerge.

The neurophysiological effects of sex steroid hormones extend far beyond reproductive functions, influencing a wide range of sensory and cognitive processes. In the olfactory system, both estradiol and progesterone exert modulatory actions through classical genomic pathways and rapid non-genomic mechanisms involving membrane-associated receptors (Lee et al., 2002; Vicini et al., 2006). These hormones interact with signaling cascades capable of altering neuronal excitability and synaptic transmission, suggesting that fluctuations in their circulating levels may directly impact olfactory processing. Indeed, behavioral and electrophysiological studies have demonstrated that olfactory sensitivity varies across the female cycle in both rodents and humans, pointing to a tight link between hormonal dynamics and odor perception (Pietras and Moulton, 1974; Kumar and Archunan, 1999). Experimental evidence further supports the presence of estradiol and progesterone receptors within key olfactory structures, including the olfactory bulb and the olfactory epithelium (Guo et al., 2001; Dillon et al., 2013; Kanageswaran et al., 2015). Activation of these receptors has been implicated in processes such as neurogenesis, plasticity, and the modulation of chemosensory input linked to social and reproductive behaviors (Pierman et al., 2008; Sanchez-Andrade and Kendrick, 2009; Maruska and Fernald, 2010). While estradiol has been more frequently studied, recent findings indicate that progesterone also plays an active role in olfactory modulation, especially via membrane progestin receptors (mPRs) expressed in olfactory receptor neurons (Pang et al., 2013; Petersen et al., 2013; Kanageswaran et al., 2015). These non-classical receptors mediate fast, hormone-dependent changes in odorant-evoked activity, as shown by electro-olfactogram and patch-clamp recordings, which revealed a rapid decrease in olfactory receptor neurons responsiveness following hormone application. Such findings strengthen the hypothesis that steroid hormones dynamically influence peripheral olfactory coding, offering a potential mechanism for hormone-dependent variability in olfactory perception (Kanageswaran et al., 2016).

These hormonal influences likely underlie the well-established sexual dimorphism of the olfactory system, reflecting structural and functional differences shaped by the organizational and activational effects of sex hormones across development and adulthood (Pierman et al., 2008; Maruska and Fernald, 2010). Animal studies have demonstrated sex-specific patterns of receptor expression, olfactory bulb volume, and neural responses to socially relevant odors, many of which are mediated by estrogen and androgen signaling (Oliveira-Pinto et al., 2014; Cherry and Baum, 2020; Abaffy et al., 2023). In humans, women consistently outperform men in tasks involving odor detection, discrimination, and identification—differences that may be partially explained by the heightened sensitivity of the female olfactory system to hormonal modulation (Cain, 1982; Doty et al., 1985; Platek et al., 2001; Doty and Cameron, 2009). Such sex-specific olfactory traits suggest an adaptive role for chemosensory cues in social and reproductive contexts and reinforce the need to account for biological sex and hormonal status when investigating sensory perception.

These sex-related differences in olfactory processing may reflect broader biological mechanisms underlying the functional importance of the olfactory system. Olfaction plays a fundamental role in human behavior and wellbeing, contributing to odor detection, flavor perception, and social interactions (Herz, 2003, 2016; Kontaris et al., 2020; Spence, 2020). The olfactory system is uniquely wired, with direct projections to limbic structures such as the amygdala and hippocampus, bypassing the thalamus and establishing strong connections with circuits involved in emotion, memory, and motivation (Mori et al., 2006; Wilson and Sullivan, 2011). This neuroanatomical organization helps explain both the emotional salience of odors and the sex-specific hormonal modulation observed in olfactory function. Olfactory dysfunctions—such as hyposmia or anosmia—are associated not only with sensory and hedonic impairments but also with altered emotional processing and increased vulnerability to psychiatric and neurodegenerative conditions (Devanand et al., 2000; Kopala et al., 2001; Croy et al., 2014; Bastos et al., 2015).

Although several studies have investigated the relationship between the menstrual cycle, oral contraceptive use, and olfactory perception, findings remain inconclusive due to considerable methodological heterogeneity. Most research relies solely on psychophysical tests, often without controlling for anatomical factors that may influence olfactory capacity. In this context, the present study aimed to evaluate olfactory performance through Sniffin’ Sticks testing at two time points aligned with hormonal variation—across the periovulatory and luteal phases in naturally cycling women, and at matched intervals during the pill regimen for contraceptive users, including continuous users. A key methodological strength was the implementation of nasofibroscopy during the screening phase, ensuring the exclusion of participants with structural alterations in the nasal cavity or olfactory region. By minimizing this often-overlooked anatomical bias, the study offers a more accurate assessment of potential hormonal effects. Participants using OCs were categorized according to hormonal formulation and dosage: low-dose (ethinylestradiol 0.02 mg) or high-dose (ethinylestradiol 0.03 mg) combined pills, estradiol valerate-based pills, and progestogen-only pills. The influence of regimen type (cyclical or continuous) and duration of use was also explored. In addition, validated scales assessing life satisfaction and subjective happiness were applied to investigate whether emotional wellbeing is associated with olfactory performance, providing a broader understanding of how hormonal and affective factors may interact to shape sensory experience.

## 2 Materials and methods

### 2.1 Subjects

A total of 98 healthy women, aged 18–40 years (28.97 ± 6.73 years), were recruited for this study. Participants were divided into two groups: 47 women who had been using an oral hormonal contraceptive (OC) for at least 4 months and up to 300 months (69.64 ± 67.13 months) and 51 women with regular menstrual cycles (non-users of hormonal contraceptives) who reported no hormonal contraceptive use for a period of at least 3 months before the study. Within the OC group, four subgroups were identified based on contraceptive composition: monophasic combined progestogen with 0.020 mg ethinyl estradiol (EE 0.02, *n* = 18), monophasic combined progestogen with 0.030 mg ethinyl estradiol (EE 0.03, *n* = 19), progestogen-only (PO, *n* = 6), and four-phase combined estradiol valerate with dienogest (EV, *n* = 4).

Exclusion criteria included the use of other hormonal contraceptives, irregular menstrual cycles, smoking, neurological disorders, endocrine diseases, autoimmune diseases, major depressive disorder, generalized anxiety disorder, long COVID-19, history of head trauma, and diagnosed anosmia, hyposmia, parosmia, or phantosmia. Additionally, participants were asked to report any medications they were currently taking as part of the standard clinical anamnesis. These reports were reviewed to identify the use of any drugs known to affect olfactory function. No participant reported using medications such as GnRH agonists (e.g., leuprorelin), antihypertensives, levothyroxine, or other agents described in the literature as capable of influencing olfactory performance. During recruitment, 15 women were excluded for the following reasons: initiation of another type of hormonal contraceptive (*n* = 4), health issues (*n* = 2), pregnancy (*n* = 1), inability to attend data collection sessions (*n* = 5), and diagnosis of an obstructive polyp during clinical examination (*n* = 3).

All participants were students or employees of Albert Einstein University, Hospital Israelita Albert Einstein, or the Centro de Ensino e Pesquisa Albert Einstein (São Paulo, Brazil). Before inclusion, they were informed about the study’s objectives and provided written informed consent. The study was approved by the Ethics Committee of the Hospital Israelita Albert Einstein and conducted in accordance with CAAE: 72180123.5.0000.0071.

### 2.2 Menstrual cycle phase determination

The menstrual cycle phase was determined based on participants’ self-reports, which were carefully monitored by the researcher (PG) throughout the study. For each participant, a spreadsheet was completed documenting the onset and duration of her menstrual cycle to identify the expected testing phase. As an additional control, participants who already used menstrual cycle tracking Apps continued to use them during data collection. Testing sessions corresponding to the periovulatory phase occurred between days 13 and 16, and those corresponding to the luteal phase occurred between days 18 and 28, before the onset of menstruation (Derntl et al., 2013; Caruso et al., 2001). All participants reported having regular menstrual cycles ranging from 28 to 32 days.

### 2.3 Clinical testing

Each participant underwent an otorhinolaryngological examination to identify any inflammatory or mechanical obstructions in the upper airway that could impair olfactory function. Nasofibroscopy, an endoscopic technique, was performed using a 3.4 mm diameter flexible fiber optic scope (Olympus), which was inserted through the nostrils, passing along the septal cartilage and lateral wall until reaching the nasopharynx. The examination also assessed the cavum region, Eustachian tubes, and Rosenmüller fossae to detect potential mechanical or infectious obstructions associated with olfactory disturbances. Participants with detected obstructions (*n* = 3) were excluded from the study.

### 2.4 Olfactory performance

Olfactory performance was assessed using the Sniffin’ Sticks Extended Test n-Butanol (Burghart Messtechnik, Wedel, Germany), a validated tool for studies with the Brazilian population (Silveira-Moriyama et al., 2008; Bastos, 2015; Trentin et al., 2022). The test kit consists of pen-like odor-dispensing devices, each approximately 14 cm long and 1.3 cm in diameter. The caps contain liquid odors or odors dissolved in propylene glycol, with a total volume of 4 ml per pen. During the test, the experimenter removed the cap for 3 s and positioned the pen tip 2 cm in front of both nostrils. To minimize external olfactory interference, the researcher wore unscented gloves and a surgical mask. Participants were instructed to avoid eating for 30 min before the test, drink only water, and refrain from wearing perfume.

The test included three assessments of olfactory function. The odor detection threshold measured the lowest concentration of a scent that could activate olfactory receptors and be perceived. This was determined using 16 sets of pens (48 in total), following a single-staircase, three-alternative, forced-choice procedure. The odor discrimination task required participants to identify the different scents in 16 sets of odor triplets. The odor identification task evaluated their ability to recognize 16 distinct odors (Rumeau et al., 2016). The Sniffin’ Sticks pens were presented 2 cm in front of the participant’s nostrils for 3–4 s, and each participant was asked to sniff at most twice following a simple verbal cue; the cap was immediately replaced after presentation (Rumeau et al., 2016).

All tests were conducted during the daytime at varying hours, in a well-ventilated room at a moderate temperature, with the absence of external odors, under standardized conditions. To ensure consistency, the same female researcher administered all assessments.

Anosmia was defined based on clinical criteria: a TDI score of 16 or lower indicated anosmia, while a score of 31 or higher indicated normosmia (Kobal et al., 2000).

### 2.5 Wellbeing scales

Subjective Happiness Scale: in this research instrument, participants characterized themselves by comparing themselves to their peers and in descriptions of happiness and unhappiness (Lyubomirsky and Lepper, 1999). Composed of 4 items where participants classify each item on a 1–7-point Likert scale, and the overall score is obtained by adding up the points for each item (Total score: 4–28 points). The Satisfaction with Life Scale: refers to the level of contentment perceived by the individual when thinking about their life in general (Diener et al., 1985). It is therefore a subjective assessment that involves weighing the level of enthusiasm/pleasure or discontentment/suffering that the individual has or feels when thinking about their way of life. Composed of 5 items where participants classify each item on a 1–7-point Likert scale, and the overall score is obtained by adding up the points for each item (Total score: 5–35 points). The two scales were translated into Portuguese and validated for the Brazilian population (Gouveia et al., 2009; Damásio et al., 2014).

### 2.6 Data collection

**FIGURE 1:**
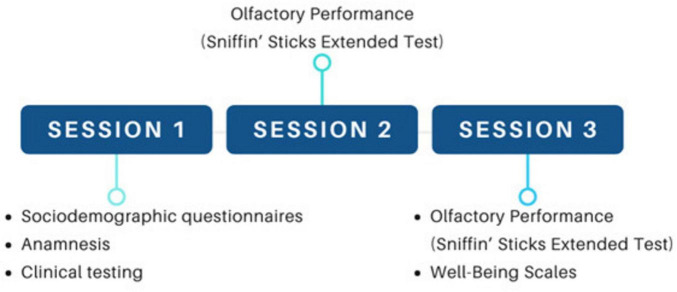
Graphic representation of the experimental design. The first session included sociodemographic questionnaires, anamnesis, and Clinical testing (Nasofibroscopy); measurement of three olfactory functions (threshold, discrimination and identification) at sessions 2 and 3; and wellbeing scales rating at session 3.

Data collection and management were conducted using the REDCap (Research Electronic Data Capture) system (Harris et al., 2009, 2019), a secure, web-based platform. Information recorded included sociodemographic characteristics, clinical history, nasofibroscopy results (classified as fit or unfit), olfactory test scores, and scores from the Subjective Happiness Scale and the Satisfaction with Life Scale. Participants were invited to attend three sessions, as illustrated schematically in Figure 1.

#### 2.6.1 Session 1

Participants completed sociodemographic and clinical questionnaires, followed by a nasofibroscopy examination performed by a trained otorhinolaryngologist to assess the integrity of the nasal cavity and olfactory region. Only participants without relevant anatomical alterations (e.g., marked septal deviation, turbinate hypertrophy) were included in subsequent testing phases.

#### 2.6.2 Session 2

Participants completed the Subjective Happiness Scale and the Satisfaction with Life Scale, followed by the first olfactory assessment using the validated Sniffin’ Sticks battery (Burghart Messtechnik GmbH, Holm, Germany), which includes threshold, discrimination, and identification (TDI) subtests. The timing of this session was determined according to the participant’s hormonal status (see below).

#### 2.6.3 Session 3

A second round of the olfactory test and the wellbeing scales was administered, scheduled to coincide with a different hormonal phase than Session 2. This design aimed to allow intra-individual comparison across phases. For women not using oral contraceptives (OCs), sessions were scheduled during the periovulatory and luteal phases, as previous research suggests differences in olfactory sensitivity across these phases (Doty and Cameron, 2009; Derntl et al., 2013). Self-reported menstrual cycle information was used to determine testing window.

Participants were randomized into different test orders to control for potential learning effects. A simple 1:1 randomization (non-stratified) was used: for non-OC users, sessions occurred either in the periovulatory phase followed by the luteal phase or in the reverse order.

For OC users, testing took place either between days 7 and 11 and subsequently between days 24 and 28 of the contraceptive regimen, or vice versa. Continuous users were also randomized regarding the order of testing sessions. The distribution of participants and testing conditions across hormonal phases is detailed in Table 1.

**TABLE 1:** Number of participants per hormonal condition and olfactory assessment phase.

Sample distribution by hormonal condition and assessment phase				
Participant group	Subgroup (OC regimen)	Session 1	Session 2	N
NOC	–	Periovulatory or Luteal	Alternate phase	51
OC	Active + hormone-free cycle	Days 7–11 or days 24–28	Alternate phase	33
	Continuous-use	Random timing	Random timing	14

Each participant completed two sessions scheduled at different hormonal phases, with session order randomized.

### 2.7 Statistical analyses

Statistical analyses were performed using R Statistical Computing Language, version 4.4.1.^1^ Descriptive statistics included mean, median, standard deviation, and interquartile range (25th and 75th percentiles) for quantitative variables, and absolute and relative frequencies for categorical variables.

To assess normality of quantitative data, the Shapiro-Wink test was applied (Shapiro and Wilk, 1965). For comparisons between two groups, the Student’s *t*-test was used when variables showed a normal distribution; otherwise, the Mann–Whitney U test was applied. Comparisons among three or more groups were performed using one-way ANOVA for normally distributed data and the Kruskal–Wallis test, followed by Dunn *post-hoc* tests, conducted with Bonferroni adjustment for multiple comparisons, using the “kwAllPairsDunnTest” function from the “PMCMRplus” package (Pohlert, 2022).

The effect sizes for the Kruskal–Wallis and Mann–Whitney tests were calculated using eta squared (η^2^) and the rank biserial correlation coefficient (r_*s*_), respectively, through the functions “kruskal_effsize” and “wilcox_effsize” from the “rstatix” package (Kassambara, 2023).

Associations between categorical variables were analyzed using the chi-square test. Correlations between olfactory performance and self-reported wellbeing, age, and duration of oral contraceptive use were assessed using Spearman’s rank correlation coefficient. Statistical significance was set at *p* < 0.05.

## 3 Results

### 3.1 Sociodemographic data

There were no significant differences between the groups using oral contraceptives (OC) and those not using them (NOC) regarding age (*p* = 0.842), household income (*p* = 0.577), or education level (*p* = 0.947), indicating sociodemographic homogeneity across groups.

### 3.2 Olfactory performance: OC vs. NOC

Descriptive statistics for the subtests and the global TDI score are presented in Table 2. The Mann–Whitney U test indicated no significant differences between the OC and NOC groups in any of the olfactory measures. Specifically, no significant differences were found for olfactory threshold (*U* = 524, *r* = 0.08, *p* = 0.262), odor discrimination (*U* = 5,156, *r* = 0.07, *p* = 0.356), odor identification (*U* = 5,172, *r* = 0.07, *p* = 0.335), or the TDI composite score (*U* = 5,312, *r* = 0.09, *p* = 0.192). The effect size r represents the rank biserial correlation coefficient. Overall, when not considering specific contraceptive compositions or menstrual cycle phases, no significant differences were observed between the OC and NOC groups.

**TABLE 2:** Descriptive data of olfactory performance measures in the two groups: women not using oral contraceptive (NOC) and women using oral contraceptive (OC).

Contraceptive using											
	NOC (not using)					OC (using)					
Test	M	MD	SD	1Q	3Q	M	MD	SD	1Q	3Q	*P*
Threshold	6.41	6.25	2.43	5.25	7.5	6.06	6	1.91	4.81	7.5	0.262
Discrimination	11.83	12	2.03	11	13	11.43	12	2.18	10	13	0.356
Identification	11.93	12	1.68	11	13	11.65	12	2.06	10	13	0.335
TDI	30.18	30.5	3.98	27.5	32.5	29.13	29.12	4.35	27.25	31.94	0.192

Mann-Whitney Test; M, Mean; MD, Median; SD, Standard Deviation; 1Q, 1st quartile; 3Q, 3rd quartile.

### 3.3 Olfactory performance by OC composition

Among OC users, four subgroups were identified based on contraceptive composition: (1) monophasic combined OC with 0.020 mg ethinylestradiol (EE) (*n* = 18), (2) monophasic combined OC with 0.030 mg EE (*n* = 19), (3) progestogen-only OC (*n* = 6), and (4) four-phase combined estradiol valerate with dienogest (*n* = 4).

The Kruskal–Wallis test revealed significant differences among these subgroups for olfactory threshold (*H* = 7.98; η^2^ = 0.05; *p* = 0.046), discrimination (*H* = 14.91; η^2^ = 0.13; *p* = 0.002), and TDI scores (*H* = 14.15; η^2^ = 0.12; *p* = 0.003), where *eta squared* (η^2^) represents the effect size (see Supplementary Table 1 for the means, medians, standard deviations, interquartile ranges, Kruskal–Wallis H values, and eta squared (η^2^) effect sizes for each olfactory test according to contraceptive composition).

Dunn’s *post-hoc* tests with Bonferroni adjustment indicated that women using valerate with dienogest showed significantly higher scores than those using 0.030 mg EE for olfactory threshold (*p* = 0.034), and higher scores than progestogen-only users for odor discrimination (*p* = 0.017) and TDI (*p* = 0.039). In addition, progestogen-only users showed lower odor discrimination (*p* = 0.011) and TDI scores (*p* = 0.043) compared with women using 0.020 mg EE (Table 3).

**TABLE 3:** The *post-hoc* Dunn test showed differences in performance on the threshold, discrimination, and TDI tests among the OC composition groups.

Oral contraceptive	Threshold	Discrimination	TDI
	P*	P*	*P* *
0.020 mg EE × 0.030 mg EE	1	0.135	0.06
0.020 mg EE × Progestogen-only	1	0.011*	0.043*
0.020 mg EE × Estradiol valerate	0.145	1	1
0.030 mg EE × Progestogen-only	1	0.751	1
0.030 mg EE × Estradiol valerate	0.034*	0.166	0.092
Progestogen-only × Estradiol valerate	0.699	0.017*	0.039*

*Dunn test. Women using Progestogen-only performed worse compared to those using 0.02 mg EE and Estradiol Valerate in the discrimination tests and TDI. The group using Estradiol Valerate performed better than the 0.03 mg group in the threshold test.

### 3.4 Olfactory performance by cycle phase

Participants were tested in two different phases according to their hormonal profile: NOC group: periovulatory and luteal phases; OC group: (days 7–11) active pill phase and (days 24–28) hormone-free interval; Continuous-use OC users: two randomized sessions without phase tracking.

The Kruskal–Wallis test indicated significant differences among the menstrual cycle phases for odor discrimination (*H* = 20.91; η^2^ = 0.09; *p* < 0.001) and TDI scores (*H* = 12.74; η^2^ = 0.04; *p* = 0.014), where eta squared (η^2^) represents the effect size [see Supplementary Table 2 for the means, medians, standard deviations, interquartile ranges, Kruskal–Wallis H values, and eta squared (η^2^)].

**FIGURE 2:**
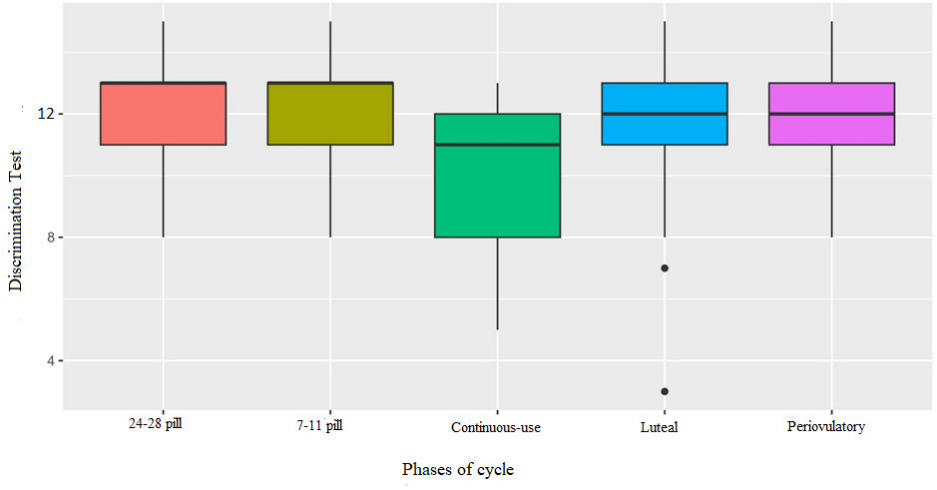
Boxplot of performance in discrimination test by cycle phases. Continuous-use OC performed worse than the 7–11 active pill phase (*p* = 0.001), the hormone-free interval 24–28 pill phase (*p* < 0.001), the periovulatory phase (*p* = 0.002), and the luteal phase (*p* = 0,004).

**FIGURE 3:**
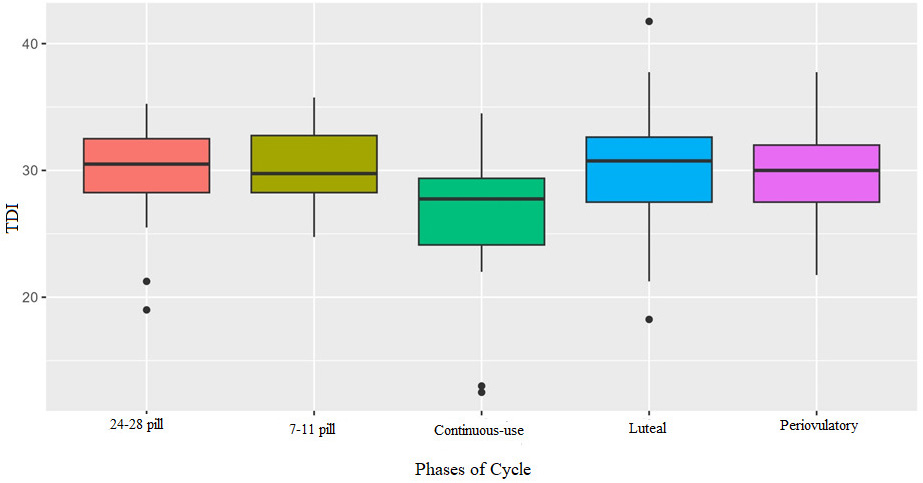
Boxplot of performance in TDI by cycle phases. Continuous-use OC showed worse performance than the hormone-free interval 24–28 pill (*p* = 0.038), and the luteal phase (*p* = 0.014).

*Post-hoc* analysis using Dunn’s test with Bonferroni correction revealed no significant differences between the standard cyclic phases (all *p* = 1). However, women using continuous OCs showed significantly lower performance on the discrimination subtest (Figure 2) and TDI (Figure 3) compared to those on cyclic regimens.

There were significant differences in odor discrimination scores between the continuous-use phase (*M* = 9.82; MD = 11; *SD* = 2.42; IQR = 4) and the active pill (*M* = 12.00; MD = 13; *SD* = 1.68; IQR = 2; *p* < 0.001), hormone-free interval (*M* = 12.00; MD = 13; *SD* = 1.70; IQR = 2; *p* = 0.001), periovulatory (*M* = 11.96; MD = 12; *SD* = 1.72; IQR = 2; *p* = 0.002), and luteal (*M* = 11.71; MD = 12; *SD* = 2.31; IQR = 2; *p* = 0.004) phases.

For the TDI score, continuous-use phase (*M* = 26.7; MD = 27.8; *SD* = 5.19; IQR = 5.3) also showed worse performance than those in the hormone-free interval (*M* = 30; MD = 31; *SD* = 3.8; IQR = 4.3; *p* = 0.038) and the luteal phase (*M* = 30.27; MD = 30.75; *SD* = 4.2; IQR = 5.1; *p* = 0.014).

### 3.5 Olfactory performance by age and the duration of OC use

The average duration of OC use was 69.64 months (range: 4–300 months). The Spearman’s correlation test revealed a negative correlation between OC duration and olfactory threshold performance (*r*_*s*_ = –0.362, *p* < 0.001), suggesting reduced sensitivity with prolonged use. No significant correlations were found for the other subtests (see Supplementary Table 3).

Among NOC participants, the Spearman’s correlation revealed a positive association between age and performance in the identification test (*r*_*s*_ = 0.217, *p* = 0.029); no significant correlation emerged for the OC group (see Supplementary Table 4).

### 3.6 Olfactory performance and wellbeing scales

Spearman’s correlation analysis revealed an association between olfactory performance and the two wellbeing scales (see Supplementary Table 5).

**FIGURE 4:**
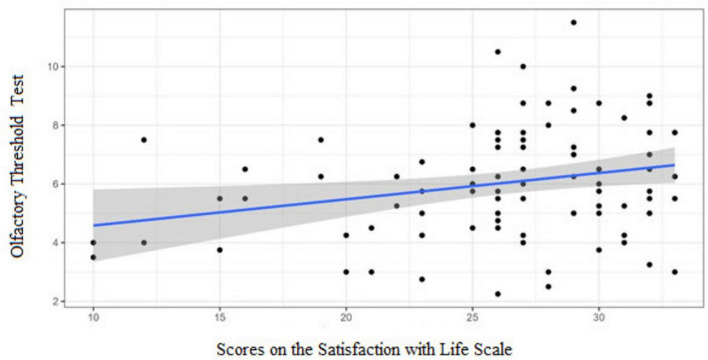
OC Group: Graphic representation of the only significant correlation between the olfactory performance (Threshold Test) of women using oral contraceptives and the Subjective Happiness Scale (*p*-value of the Spearman’s correlation = 0.026).

In the OC group, higher olfactory threshold scores were positively correlated with greater life satisfaction (*r*_*s*_ = 0.229; *p* = 0.026) (Figure 4). No significant correlations were found for the other subtests.

**FIGURE 5:**
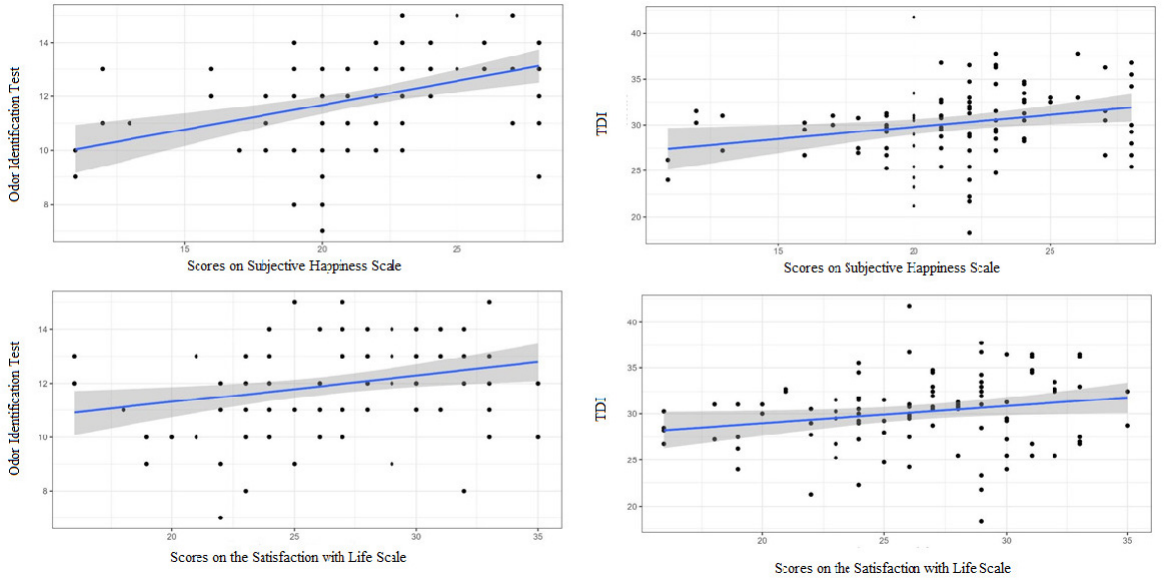
NOC group: graphic representation of the correlations between the olfactory performance of women not using oral contraceptives and the wellbeing Scales. Higher scores on the odor identification test are associated with greater self-reported happiness (r_s_ = 0.486, *p* < 0.001) and life satisfaction (r_s_ = 0.283, *p* = 0.004). Higher score on the TDI was also linked to higher scores on the Subjective Happiness Scale (*r*_s_ = 0.301, *p* = 0.002) and the Satisfaction with Life Scale (*r*_s_ = 0.232, *p* = 0.019). Spearman’s correlation test.

In the NOC group, identification and TDI scores were positively associated with both wellbeing scales (Figure 5). Specifically, odor identification correlated with subjective happiness (*r*_s_ = 0.486; *p* < 0.001) and life satisfaction (*r*_s_ = 0.283; *p* = 0.004); TDI scores correlated with subjective happiness (*r*_s_ = 0.301; *p* = 0.002) and life satisfaction (*r*_s_ = 0.232; *p* = 0.019). No significant correlations were observed for threshold or discrimination in this group.

## 4 Discussion

This study aimed to investigate the influence of OC intake on olfactory performance, expanding the current understanding of hormonal modulation of the sense of smell, particularly in relation to OC type, dosage, usage duration, and wellbeing. Despite extensive literature on the topic, results across studies remain inconsistent, possibly due to differences in methodological design, odor types used, and population characteristics.

In our sample, no significant differences were observed in the general olfactory performance of OC users and non-users, aligning with previous findings (Schaefer et al., 2021). However, contrasting results in the literature emphasize that hormonal influences on olfaction may depend on the specific odorant tested (Lundström et al., 2006; Doty and Cameron, 2009). For instance, Caruso et al. (2001) found that OC users presented distinct thresholds compared to naturally cycling women during the periovulatory and follicular phases, with similar sensitivity to the luteal phase. These findings suggest that OC use may stabilize hormonal fluctuations but does not uniformly enhance or impair olfactory abilities.

Interestingly, while no general effect was found for OC use, our results suggested differences in olfactory performance across contraceptive formulations. Women taking four-phase combined estradiol valerate showed higher olfactory threshold and overall TDI scores compared to users of other formulations. This pattern is consistent with evidence suggesting that estradiol may facilitate olfactory sensitivity through its action on central and peripheral olfactory pathways. Estradiol has been shown to influence olfactory function through both peripheral and central pathways. In rodents, estradiol treatment increases astrocyte density, olfactory epithelium thickness, and the number of mature olfactory neurons (Abaffy et al., 2023). The olfactory bulb is a known substrate for sex steroid modulation, with reproductive states altering the expression of estrogen receptors in this region (Cole and Stacey, 2006). Aromatase, the enzyme responsible for the conversion of androgens to estrogens, is expressed in central olfactory areas such as the piriform cortex and olfactory bulb and has been implicated in reproductive behavior and olfactory memory (Luine and Dohanich, 2008; Stanić et al., 2021). Estradiol also enhances dopaminergic activity by increasing tyrosine hydroxylase expression, which is relevant since dopamine plays a role in olfactory processing and memory (Frye et al., 2007; Liu et al., 2008; Stanić et al., 2021). Importantly, pharmacokinetic differences between estradiol valerate and ethinylestradiol (EE) may underlie these effects. Estradiol valerate is a prodrug of 17β-estradiol, the primary endogenous estrogen, and once metabolized, it binds to estrogen receptors (ERα and ERβ) with natural affinity and a physiological activation pattern. In contrast, EE contains an ethinyl group at the C17 position, which extends its half-life but also results in disproportionately strong and prolonged activation of ERα and a weaker effect on ERβ, leading to altered receptor signaling (Sitruk-Ware and Nath, 2013; Stanczyk et al., 2013; Hampson, 2023). These receptor-binding differences may affect downstream modulation of neuroplasticity and sensory processing. Furthermore, alterations in estrogen receptor distribution and density have been observed in the nasal mucosa across the menstrual cycle and in OC users, potentially affecting peripheral olfactory processing (Kollndorfer et al., 2016; Derntl et al., 2020). EE, At higher doses, EE may interfere with endogenous estrogen feedback mechanisms and disrupt natural receptor regeneration cycles, which could lead to reduced olfactory sensitivity over time (Genazzani et al., 2023). These findings collectively support the hypothesis that formulations based on natural estrogens like estradiol valerate may exert more physiological effects on olfactory-related circuits than synthetic EE, although larger samples are needed to confirm this potential advantage. However, this finding should be interpreted with caution, as it is based on a very small subgroup (*n* = 4) and therefore must be regarded as exploratory. Rather than indicating a definitive effect of estradiol valerate, these results point to a potential association that warrants further investigation in larger and more balanced samples.

Conversely, users of progestogen-only pills exhibited worse olfactory performance than those using combined oral contraceptives with low-dose EE, a finding that may reflect the differential influence of specific progestins on olfactory signaling pathways. Nevertheless, given the limited number of participants in this subgroup (*n* = 6), these findings should be interpreted as preliminary. They do not allow conclusions about the effects of progestogens as a class, but rather suggest that different hormonal profiles and exposure patterns may be associated with variability in olfactory function. Animal studies have demonstrated progesterone’s capacity to reduce sensitivity in the olfactory epithelium and decrease activity in the olfactory bulb (Dey et al., 2015; Kanageswaran et al., 2016). At a neurobiological level, different progestogens exert distinct effects on neuronal function, which may contribute to these differences in performance. For instance, medroxyprogesterone acetate (MPA), a synthetic progestin with known antagonistic activity on estrogen receptors (ERα and ERβ), has been shown to block the neuroprotective actions of estradiol by downregulating these receptors (Brinton et al., 2008; Jayaraman and Pike, 2014). This disruption in estrogen signaling can impair neuroplasticity and olfactory processing, particularly in brain regions associated with sensory integration. Moreover, the modulation of brain-derived neurotrophic factor (BDNF)—a key molecule for synaptic plasticity and olfactory learning—appears to be progestin-specific. While compounds such as levonorgestrel and nesterone have been linked to increased BDNF expression, MPA has been associated with reduced BDNF levels, indicating divergent impacts on neuronal resilience and cognitive-sensory function (Jayaraman and Pike, 2014). These findings align with our observation that progestogen-only users, likely exposed to less neuroprotective progestins, performed more poorly than those using combined formulations. Additionally, the structural variation among progestogens influences their agonistic or antagonistic behavior on progesterone and estrogen receptors, which in turn affects apoptosis regulation and neuroinflammation. Some progestins may upregulate caspase activity and pro-inflammatory cytokines, contributing to neuronal vulnerability, while others exert anti-apoptotic, neuroprotective effects when combined with estrogens (Arevalo et al., 2015; Brinton, 2004). Thus, the heterogeneity in olfactory performance observed among OC users may reflect these underlying neuroendocrine mechanisms rather than the EE dosage alone. The lack of significant differences between the 0.02 and 0.03 mg EE groups supports prior findings (Kollndorfer et al., 2016), suggesting that estrogen dose *per se* is not the primary driver, and that the type and action profile of the progestin component warrant closer scrutiny in future studies.

Regarding hormonal cycle phases, we found no consistent differences in olfactory performance across the different stages of OC intake or in non-users’ natural cycles. However, continuous OC use was associated with significantly lower performance in discrimination and overall TDI scores. While studies such as Derntl et al. (2013) have reported phase-specific differences in naturally cycling women—particularly enhanced sensitivity during periovulatory periods—such effects appear to be more evident for specific odorants, especially social odors. Studies have shown that ovulating women demonstrate higher sensitivity to androstadienone, androstenone, and other putative pheromones during fertile phases, while OC users show reduced discrimination of these social chemosignals (Lundström et al., 2003; Endevelt-Shapira et al., 2020). The lack of differences in our study may stem from the exclusive use of common environmental odors in the Sniffin’ Sticks test, rather than socially or hormonally relevant compounds. Still, it is important to emphasize that the Sniffin’ Sticks Extended Test is a gold-standard, extensively validated method that offers a comprehensive assessment of olfactory function. By measuring threshold, discrimination, and identification, it captures both sensory sensitivity and higher-order cognitive processing, ensuring a robust and standardized evaluation of olfactory capacity that remains highly informative in clinical and research contexts (Hummel et al., 1997).

Importantly, we did not assess hedonic valence, which is modulated by hormonal states and may reveal additional perceptual changes. Research shows that sex hormones influence affective responses to odors, possibly via their effects on brain regions such as the amygdala, orbitofrontal cortex, and hypothalamus (Derntl et al., 2008; Dreher et al., 2007; Goldstein et al., 2005). Estrogen enhances amygdala reactivity to emotional stimuli (Goldstein et al., 2005), while progesterone affects limbic-prefrontal connectivity (Gingnell et al., 2012). Moreover, hedonic perception is modulated by dopaminergic reward pathways (Berridge and Kringelbach, 2015). Future studies should integrate hedonic evaluations to explore the intersection of hormonal status, emotion, and olfactory experience more comprehensively.

Correlational analyses showed a negative association between OC duration and olfactory threshold performance, diverging from studies reporting positive associations with identification ability (Derntl et al., 2013) or no association (Kollndorfer et al., 2016). Among non-users, age was positively correlated with identification performance, potentially reflecting accumulated olfactory learning—a pattern absent among OC users. Identification relies on verbal memory and semantic knowledge (Larsson et al., 2000; Hedner et al., 2010), which develop through contextual odor exposure (Rabin, 1988; Lehrner et al., 1999), and are supported by hippocampal-piriform interactions (Wilson, 2003). The absence of this effect in OC users may suggest interference with typical olfactory-cognitive trajectories, possibly via neuroplastic changes in memory-sensitive regions like the orbitofrontal cortex and hippocampus (Jacobs and D’Esposito, 2011; Pletzer et al., 2019). These results highlight the broader impact of hormonal modulation not only on sensory perception but also on cognitive-emotional integration.

Finally, a notable finding was the association between olfactory performance and self-reported wellbeing. In OC group, higher threshold scores were positively correlated with greater life satisfaction, whereas in the NOC group, odor identification and overall TDI scores were positively correlated with both subjective happiness and life satisfaction. This aligns with literature linking olfaction and emotion (Herz, 2016; Kontaris et al., 2020). Olfactory input projects directly to the amygdala and orbitofrontal cortex, bypassing the thalamus and enhancing emotional salience (Zald and Pardo, 1997; Royet et al., 2003). Olfactory dysfunction has been associated with affective disorders, while positive emotional states enhance odor perception and memory (Croy et al., 2014; Seubert et al., 2009). Mood disorders and stress, in contrast, reduce olfactory sensitivity (Pause et al., 2001). Structural studies also reveal differences between OC users and non-users in regions involved in both olfaction and affect, including the amygdala, hippocampus, and salience networks (Lisofsky et al., 2015), suggesting neuroanatomical bases for these associations.

One limitation of the present study concerns the sample size and its distribution across groups. Although the total number of participants (*N* = 98) provided sufficient power for the main analyses, the subgroup sizes within the oral contraceptive users were relatively small and unbalanced (e.g., estradiol valerate with dienogest and progestin-only users). These small subgroups limit the statistical power and the extent to which the findings can be generalized. Therefore, these results should be interpreted as preliminary and exploratory, highlighting the need for future studies with larger and more balanced samples to confirm and expand upon our observations.

Another relevant limitation of this study is the lack of direct quantification of serum estrogen and progesterone levels. Although hormone concentrations were not measured, the classification of menstrual cycle phases in non-users relied on self-reported tracking and the estimated timing of ovulation, a procedure frequently employed in olfactory research with reliable outcomes (e.g., Derntl et al., 2013; Lundström et al., 2006). While this introduces a degree of variability, it does not invalidate the present findings, which remain consistent with the existing literature. Nevertheless, future studies would benefit from the inclusion of hormonal assays to better establish dose-dependent relationships between olfactory performance and circulating levels of sex hormones. Hormonal fluctuations not only vary across the menstrual cycle and between individuals, but may also differ by ethnicity. For instance, estrogen levels have been shown to be significantly higher across the menstrual cycle in African-American women compared to Caucasian women (Marsh et al., 2011), and distinct hormonal patterns have also been reported during the menopausal transition (Randolph et al., 2003; Setiawan et al., 2006). This additional layer of biological variability is essential for refining our understanding of how sex hormones modulate olfactory processing.

Although the present study relied on psychophysical measures of olfactory performance and self-reported emotional wellbeing, recent research has increasingly emphasized the value of complementing behavioral assessments with physiological approaches to sensory processing. Techniques such as electrophysiological recordings (Sowndhararajan and Kim, 2016; Yao et al., 2024; Wang et al., 2025), neuroimaging methods (Kulason et al., 2022; Gunasekara et al., 2022), and autonomic measures (Bensafi et al., 2002; Jung et al., 2013; Fukada et al., 2012) can provide direct insights into the neural and peripheral mechanisms underlying olfactory perception and its modulation by hormonal factors. Integrating psychophysical and physiological assessments in future investigations may help clarify the biological pathways through which hormonal contraceptives and menstrual cycle phases influence olfactory function and its relationship with emotional wellbeing.

In summary, the present study did not reveal overall differences in olfactory performance between users and non-users of oral contraceptives. However, exploratory analyses suggest that olfactory performance may vary according to contraceptive formulation, regimen, duration of use, and subjective wellbeing. Importantly, these associations should be interpreted with caution, given the relatively small and uneven subgroup sizes, as well as the absence of direct hormonal measurements. Rather than demonstrating definitive effects, our findings highlight underexplored relationships between hormonal modulation, sensory processing, and emotional factors that warrant further investigation. Future studies combining psychophysical assessments with physiological and neurobiological measures, larger and more balanced samples, and direct hormone quantification will be essential to clarify the mechanisms by which sex hormones and contraceptive formulations may influence olfactory function.

## Data Availability

The raw data supporting the conclusions of this article will be made available by the authors, without undue reservation.

## Footnotes

1
https://www.R-project.org/
