# Supplementary material for: Olfactory perception and wellbeing across hormonal contraceptive users and menstrual cycle phases
Source: Front Hum Neurosci. 2026 Feb 3;20:1646597. doi: 10.3389/fnhum.2026.1646597 (PMC12909581; doi:10.3389/fnhum.2026.1646597)
Supplement: Supplementary file 1 [file Data_Sheet_1.pdf]

Supplementary Materials

Table S1. Descriptive statistics of olfactory performance according to the composition of the contraceptive.

Test	Contraceptive composition																		
	0.020mg EE				0.030mg EE				Progestogen-only				Estradiol valerate						
	M	MD	SD	IIQ	M	MD	SD	IIQ	M	MD	SD	IIQ	M	MD	SD	IIQ	H	p*	η²
Threshold	6,1	5,6	1,8	2,1	5,6	5,6	2,06	2,94	6,23	6,12	1,25	1	7,62	7,75	1,66	1,4	7,98	0,046	0,055
Discrimination	12	13	1,5	2	11	12	2,34	4	9,67	10	2,57	4,3	12,75	13	1,04	1,3	14,91	0,002	0,132
Identification	12	13	2,1	3	11	12	2,18	2,75	11,33	11	1,78	2,3	11,25	11,5	1,49	2,3	3,82	0,282	0,009
TDI	31	31	3,6	4,6	28	28	5,09	5,88	27,23	28	3,07	3,8	31,62	31,38	1,98	1,6	14,15	0,003	0,124

*Kruskal-Wallis Test; M = Mean; MD = Median; SD = standard deviation; IIQ = interquartile range; Kruskal-Wallis H; p-value; η² = effect size

Table S2. Descriptive statistics of olfactory performance according to the phase of the cycle.

Test	Cycle phase																						
	Hormone-free interval				Active pill phase				Continuous use				Luteal				Periovulatory						
	M	MD	SD	IIQ	M	MD	SD	IIQ	M	MD	SD	IIQ	M	MD	SD	IIQ	M	MD	SD	IIQ	H	p*	η²
Threshold	6,4	6,3	1,8	1,8	6,4	6,5	2,11	3	5,34	5,5	1,63	2,1	6,49	6,5	2,32	2	6,33	6,25	2,55	2,9	6,64	0,156	0,014
Discrimination	12	13	1,7	2	12	13	1,68	2	9,82	11	2,42	4	11,71	12	2,31	2	11,96	12	1,72	2	20,91	<0,001	0,089
Identification	12	12	2	3	12	12	1,76	2	11,54	11,5	2,49	2,3	12,08	12	1,8	2,5	11,78	12	1,57	2	2,46	0,651	-0,008
TDI	30	31	3,8	4,3	30	30	3,2	4,5	26,7	27,8	5,19	5,3	30,27	30,75	4,2	5,1	30,08	30	3,8	4,5	12,46	0,014	0,044

*Kruskal-Wallis Test; M = Mean; MD = Median; SD = standard deviation; IIQ = interquartile range; Kruskal-Wallis H; p-value; η² = effect size

Table S3. Correlations for OC usage time and olfactory performance

Test	Duration of OC use	
	r_s	p-value
Threshold	-0,362	<0,001
Discrimination	-0,114	0,272
Identification	-0,023	0,824
TDI	-0,198	0,056

rs= Spearman's rank correlation coefficient

Table S4. Correlations for age and olfactory performance

	Age			
	OC		NOC	
Test	r_s	p-value	r_s	p-value
Threshold	-0,107	0,303	-0,082	0,415
Discrimination	-0,194	0,061	0,134	0,178
Identification	0,067	0,524	0,217	0,029
TDI	-0,127	0,222	0,08	0,425

rs= Spearman's rank correlation coefficient

Table S5. Correlations between well-being scales and olfactory performance for users (OC) and non-users (NOC) of oral contraceptives.

Test	OC				NOC			
	Subjective Happiness Scale		Life Satisfaction Scale		Subjective Happiness Scale		Life Satisfaction Scale	
	r_s	p-value	r_s	p-value	r_s	p-value	r_s	p-value
Threshold	0,138	0,186	0,229	0,026	0,07	0,482	0,078	0,438
Discrimination	-0,1	0,338	0,015	0,889	0,118	0,238	0,106	0,29
Identification	-0,166	0,11	-0,103	0,324	0,486	<0,001	0,283	0,004
TDI	-0,059	0,572	0,099	0,341	0,301	0,002	0,232	0,019

rs= Spearman's rank correlation coefficient
